# Multi-omics reveals that streptomycin sulfate induces obesity in *Halyomorpha halys* by disrupting the gut microbiome-metabolome axis

**DOI:** 10.1016/j.isci.2026.116612

**Published:** 2026-06-30

**Authors:** Xiaoyu Yan, Yaquan Lv, Dianyu Liu, Yu Chen, Zhihan Su, Wenyan Xu, Xiaolin Dong, Chenxi Liu

**Affiliations:** 1 College of Agriculture, Yangtze University, No. 1 Nanhuan Road, Jingzhou 434025, China; 2 Sino-American Biological Control Laboratory, Institute of Plant Protection, Chinese Academy of Agricultural Sciences, No. 2 Yuanmingyuan West Road, Haidian District, Beijing 100193, China

**Keywords:** Streptomycin Sulfate, Obesity, *Halyomorpha halys*, metabolic disease

## Abstract

The global obesity epidemic is linked to antibiotic exposure, yet mechanisms remain unclear. We found that streptomycin sulfate (SM) exposure induced obesity in *Halyomorpha halys*. Using a multi-omics approach (16S rRNA sequencing, metabolomics, and transcriptomics), we demonstrated that SM restructured the gut microbiome in a sex-specific manner and dysregulated key metabolic pathways. Integrated analysis revealed a robust network linking altered gut bacteria, disrupted metabolites, and host transcriptional responses. Our findings establish that SM promotes obesity by disrupting the gut microbiome-metabolome interface, providing mechanistic insights into antibiotic-induced metabolic dysfunction.

## Highlights

• Streptomycin sulfate (SM) induces obesity in *Halyomorpha halys*
• SM disrupts the gut microbiome in a sex-specific manner
• Multi-omics reveals a disrupted microbiome-metabolome axis
• This study provides mechanistic insights into antibiotic-induced metabolic dysfunction

## Graphical abstract

**Figure:**
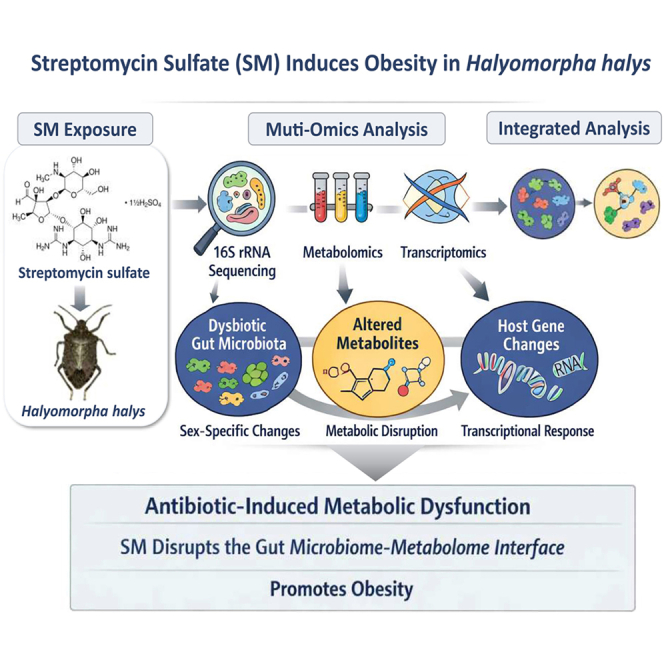

## Teaser

Microbiome; Metabolomics; Transcriptomics

## Introduction

Since 1975, the incidence of obesity has nearly tripled, presenting considerable health hazards and societal challenges.^1^ This rising prevalence has fueled a strong desire to understand its etiology. The impact of antibiotics on promoting weight gain in livestock and humans has been reported since the 1940s, drawing considerable attention to their potential contribution to the obesity epidemic.^2^^,^^3^ In 2005, Ternak first proposed that low-dose antibiotic exposure in humans might lead to weight gain.^4^ Several hypotheses have been proposed to explain the mechanisms underlying antibiotic-induced weight gain in animals: (1) stimulating the growth of gut bacteria that synthesize essential nutrients; (2) enhancing intestinal absorption of nutrients; (3) inhibiting microorganisms that compete with the host for nutrients; and (4) suppressing subclinical infections and overt diseases, thereby improving overall animal health and nutritional status.^5^ These mechanisms may collectively alter energy intake and metabolism, promoting weight gain in the host.

Streptomycin sulfate (SM), an aminoglycoside antibiotic, inhibits protein synthesis.^6^ It can kill or inhibit the growth of *Mycobacterium tuberculosis* and exhibits potent antibacterial activity against various Gram-positive and Gram-negative bacteria.^7^ SM is widely used as an antibacterial agent in human therapy, animal husbandry, and agriculture.^8^ Studies have reported that adding low-dose SM and sulfapyridine to chicken feed increases the weight of chicks.^9^

In recent years, growing research has focused on the gut microbiota as a key mediator of antibiotic-induced metabolic effects in the host. Accumulating evidence has confirmed that the compositional characteristics of gut microbiota in mice are correlated with metabolite alterations across multiple tissues, and are closely associated with lipid metabolism processes, thereby regulating systemic metabolic activities.^10^ The gut microbiota can also modulate intestinal barrier function and host immune status, which in turn affects the progression of glucose and lipid metabolism in the body. Furthermore, it regulates the enteroendocrine system by influencing the secretion of hormones such as GLP-1 and PYY, which are directly involved in glucose homeostasis and lipid metabolism regulation.^11^^,^^12^ Existing studies have demonstrated that gut microbiota alterations can lead to metabolic endotoxemia through two interrelated mechanisms: increased intestinal permeability (“leaky gut”) that allows more LPS to translocate into circulation, and shifts in the abundance of LPS-producing Gram-negative bacteria.^13^^,^^14^ A team led by JI Gordon investigated gut microbiota as an environmental factor that regulates fat storage in obesity. They demonstrated that the transfer of intestinal bacteria from obese mice to germ-free ones resulted in heightened fat accumulation in the recipients.^15^ Exposure of *Drosophila melanogaster* to sulfamethoxazole increases the proportion of Firmicutes in the gut microbiota, disrupts circadian rhythms and lipid metabolism, and leads to weight gain.^16^ The increased body weight and fat mass were not associated with increased food intake, but with enhanced food conversion efficiency.^17^ Bacterial metabolites such as short-chain fatty acids (SCFAs) can regulate appetite, insulin signaling, and adipogenic processes; thus, the gut microbiota can modulate the host to store lipids in adipose tissue, leading to the development of obesity. This phenomenon has been repeatedly observed in mice and confirmed in *Drosophila melanogaster*.^18^

Obesity is a complex disease arising from the interplay of multiple factors, including genetic susceptibility, environmental triggers, and physiological metabolic disruptions. This complexity makes it challenging to establish an ideal model for analyzing the underlying genetic and physiological mechanisms—a difficulty that extends to the functional characterization of various compounds and the systematic investigation of signaling pathways. Currently, insects are widely recognized by the academic community as valuable and versatile experimental models for studying complex diseases. Their core advantages include a short life cycle, low breeding costs, the capacity to produce a large number of offspring in a short period, and fewer ethical concerns. Furthermore, recent breakthroughs in insect genomics and the continued development and refinement of genetic research methodologies have further promoted the widespread application of insects in fundamental physiology, biochemistry, genetics, and molecular biology.^19^^,^^20^ Despite fundamental differences in morphology and anatomy between insect and mammalian systems, the pathways involved in developing metabolic abnormalities may be evolutionarily conserved. The structural similarities, as well as conserved patterns in gene expression, regulatory mechanisms of gene activity, signaling pathway processes, intercellular transport, and mechanisms of cell death induction, collectively provide the rationale for using insects as research models for human diseases.^19^^,^^21^ Multiple homologs of human obesity-related genes have been identified in insects, including GIPR, TUB, and FAIM2. The vast majority of these genes possess homologs in distantly related species, such as *Drosophila melanogaster* and *Caenorhabditis elegans*.^22^ Insect models have demonstrated potential in evaluating the efficacy of antidiabetic drugs. Furthermore, their capacity to analyze disease symptoms and correlate them with their mammalian counterparts suggests that insects can serve as experimental models for studying human metabolic diseases and their underlying mechanisms.^23^^,^^24^

*Halyomorpha halys* is a major agricultural and forestry pest with a long lifespan in both adult and nymph stages, causing damage over an extended period.^25^ It infests over 300 host species, including many economically important crops, and is particularly harmful to fruit trees such as peaches, pears, and apples.^26^ The weight of *H. halys* increases significantly under suitable environmental conditions with adequate food and water supply, stabilizing when it reaches adulthood. *H. halys* harbors a specific symbiotic bacterium, *Pantoea carbekii*, which synthesizes multiple essential amino acids and is crucial for host growth and development.^27^ In this study, we regulated the diet and water availability of adult *H. halys* with SM for 14 days to investigate weight gain in male and female individuals. Using multi-omics approaches, including metabolomics, 16S rRNA high-throughput sequencing, and transcriptomics, we aimed to investigate the obesity-inducing effects of SM on *H. halys* and its molecular basis, providing insights into the potential mechanisms of insect obesity.

## Results

### Effects of SM on the health of *H. halys*

**Figure 1:**
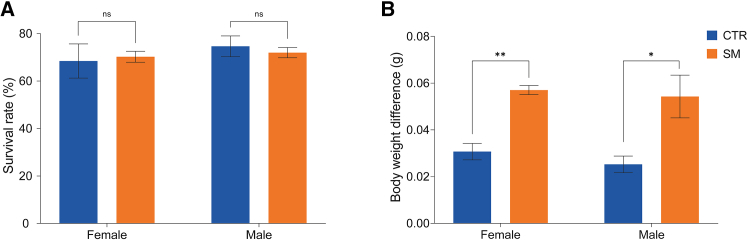
Effects of SM on the survival rate and body weight of *H. halys* of different sexes (A) Survival rate of *H. halys* over 14 days of SM treatment (1.0 g/L) (*n* = 200 individuals per sex per group). (B) Body weight gain of female and male *H. halys* after 14 days of SM treatment (*n* = 200 individuals per sex per group). Data are represented as mean ± SEM. Statistical significance was determined using an independent samples *t* test. ∗*p* < 0.05 and ∗∗*p* < 0.01; ns, not significant. CTR: control group. SM: streptomycin sulfate-treated group.

The potential impact of SM treatment on the body weight and survival rate of *H. halys* was investigated. The results showed that males and females exhibited significantly greater weight gain when *H. halys* were fed corn treated with 1.0 g/L SM alongside water (*p* < 0.05) than the control group. Specifically, the average body weight of female *H. halys* in the treatment group increased by 57.03 mg, while that of males increased by 54.27 mg (Figure 1B), whereas the average body weight gain of females and males in the control group was 30.70 mg and 25.23 mg, respectively. However, SM treatment had no effect on the survival rate of *H. halys* (*p* > 0.05, Figure 1A). Additionally, SM treatments at the concentrations of 0.1 g/L and 0.6 g/L showed no significant impacts on either the body weight or survival rate of *H. halys* (Figure S1). This result suggests that SM may disrupt the metabolism of *H. halys*, which in turn leads to increased weight gain.

### SM induced metabolite changes in *H. halys*

**Figure 2:**
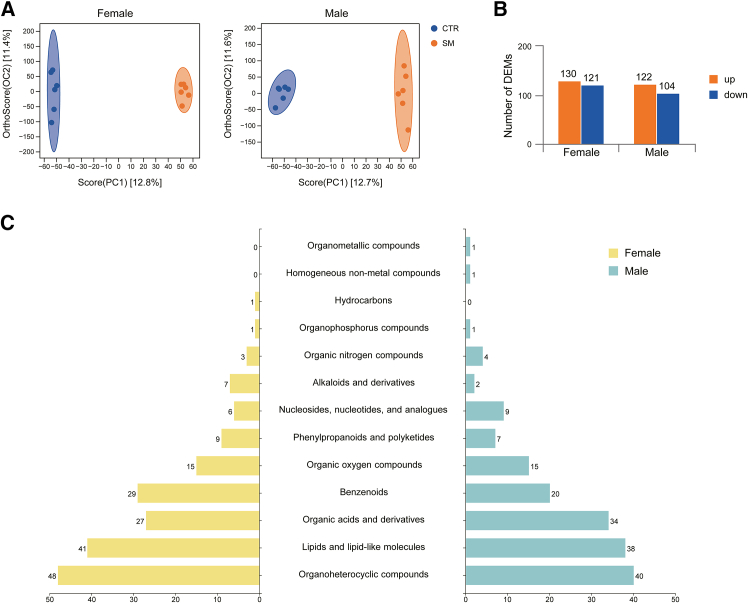
SM-induced metabolomic alteration in *H. halys* (A) OPLS-DA scatterplot displays the patterns of metabolite changes between the control and SM groups (*n* = 6 biological replicates per group). (B) The number of differentially expressed metabolites (DEMs) between the control and SM groups. (C) The interactive pyramid plot shows classification and numbers of DEMs in male and female *H. halys* under positive and negative ion modes.

Untargeted metabolomic analysis was conducted to assess the metabolite profiles of *H. halys* in each treatment group and explore the general biological responses to SM. We used an OPLS-DA model to visualize metabolite profiles of the control and antibiotic groups (Figure 2A). The results showed that the male and female antibiotic groups were significantly separated from the control group, with marked differences in their metabolites, suggesting that the metabolism of *H. halys* is highly sensitive to SM. Our untargeted metabolomics analysis successfully annotated 1,735 metabolites, comprising 1,022 in positive ion mode and 713 in negative ion mode (Tables S2 and S3).

Variables with VIP >1 and *p* < 0.05 were identified as differentially expressed metabolites (DEMs). When detected in both positive and negative ion modes, 226 (up = 122, down = 104), and 251 (up = 130, down = 121) DEMs were observed in male and female *H. halys*, respectively (Figure 2B). The identified DEMs in female insects mainly consist of organic heterocyclic compounds (48), lipids and lipid-like molecules (41), benzenoids (29), organic acids and derivatives (27), organic oxygen compounds (15), phenylpropanoids and polyketides (9), alkaloids and derivatives (7), nucleosides, nucleotides, and analogues (6) and organic nitrogen compounds (3). In contrast, those in male insects include organoheterocyclic compounds (40), lipids and lipid-like molecules (38), organic acids and derivatives (34), benzenoids (20), organic oxygen compounds (15), nucleosides, nucleotides, and analogs (9), phenylpropanoids and polyketides (7), and organic nitrogen compounds (4) (Figure 2C).

**Figure 3:**
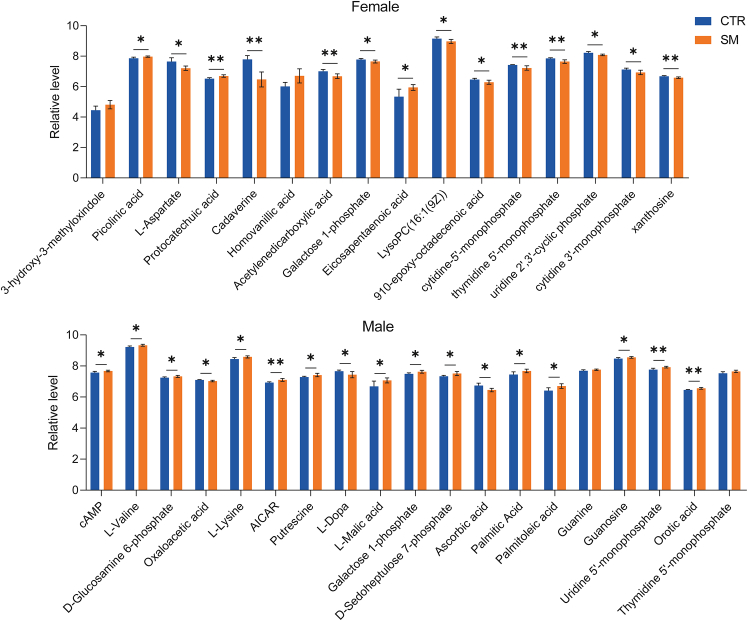
The effect of SM on substances involved in the metabolism of amino acids, carbohydrates, lipids, and nucleosides Data are represented as mean ± SEM. Statistical significance was determined using an independent samples *t* test. ∗*p* < 0.05 and ∗∗*p* < 0.01.

We determined the functions of DEMs using the KEGG pathway analysis. Feeding with SM disrupted the regulation of genetic and environmental information processing (Tables S4 and S5). Feeding female *H. halys* with SM significantly affected nucleotide metabolism (pyrimidine metabolism), metabolism of other amino acids (D-amino acid metabolism), and lipid metabolism (linoleic acid metabolism and glycerophospholipid metabolism) (*p* < 0.05). Disruption of lipid metabolic pathways in the antibiotic group led to upregulated levels of eicosapentaenoic acid, alongside downregulated levels of lysoPC (16:1(9Z)). Perturbation of amino acid pathways upregulated picolinic acid and homovanillic acid while downregulating L-aspartate and cadaverine. In carbohydrate metabolism, acetylenedicarboxylic acid and galactose 1-phosphate were downregulated. Alterations in purine and pyrimidine pathways downregulated cytidine-5′-monophosphate, thymidine 5′-monophosphate, uridine 2′,3′-cyclic phosphate, cytidine 3′-monophosphate, and xanthosine (Figure 3).

Feeding male *H. halys* with SM significantly affected amino acid metabolism (valine, leucine and isoleucine biosynthesis and alanine, aspartate and glutamate metabolism), carbohydrate metabolism (TCA cycle and pyruvate metabolism), global and overview maps (biosynthesis of amino acids, biosynthesis of cofactors, metabolic pathways and 2-oxocarboxylic acid metabolism), metabolism of cofactors and vitamins (pantothenate and CoA biosynthesis and biotin metabolism), metabolism of other amino acids (D-amino acid metabolism and glutathione metabolism, nucleotide metabolism (purine metabolism and pyrimidine metabolism) (*p* < 0.05). Changes in lipid metabolism pathways upregulated palmitic acid and palmitoleic acid while downregulating farnesyl pyrophosphate. Perturbation of amino acid pathways upregulated L-valine, L-lysine, D-Glucosamine 6-phosphate, AICAR, and putrescine while downregulating oxaloacetic acid and L-DOPA. Upregulated metabolites in the carbohydrate metabolism pathway include L-malic acid and galactose 1-phosphate, whereas downregulated ones include oxaloacetic acid and ascorbic acid. In nucleotide metabolism, guanosine, uridine 5′-monophosphate, thymidine 5′-monophosphate, and orotic acid were upregulated (Figure 3). These metabolites are critical components of biological processes such as homeostasis and energy metabolism, and perturbations in their levels can directly or indirectly affect host phenotypes.

### Effects of SM on the *H. halys* gut bacterial community

**Figure 4:**
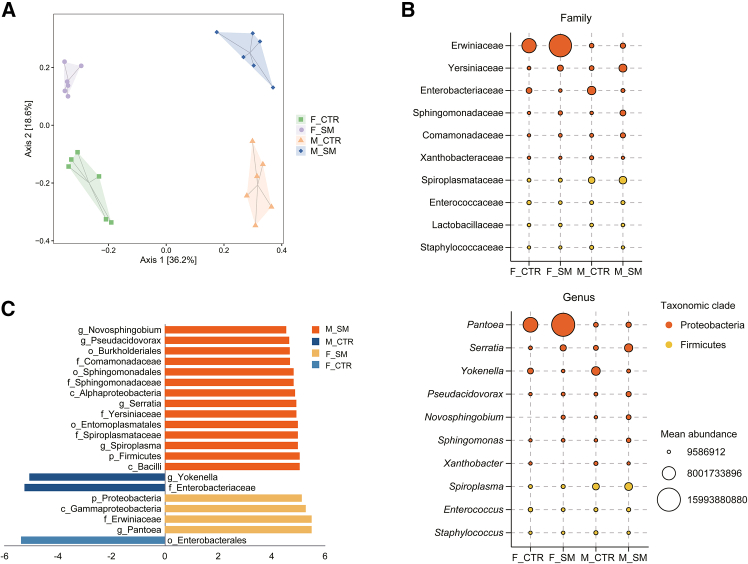
Effects of SM on gut microbiota structure and diversity in *H. halys* (A) Principal coordinate analysis (PCoA) plot based on Bray-Curtis distance matrix visualizes the gut bacterial community structure of male and female *H. halys* in the control and SM groups (*n* = 6 biological replicates per group, with 40 guts per replicate). (B) Bubble plots based on the family and genus taxa show the differences between the control and SM groups. (C) Differentially abundant bacteria between the control and SM groups were analyzed using LEFSe analysis with different haze levels. Linear discriminant analysis (LDA) scores indicate the degree of consistent difference in relative abundance between the control and SM groups; log LDA score of >4.5 was used for the analysis.

High-throughput sequencing was performed to examine alterations in the gut microbiome of *H. halys* fed with SM. Analysis of the 16S rRNA amplicon sequences from gut samples of four groups of *H. halys* yielded 3,282,639 raw tags and 2,816,105 valid tags (Table S6). Based on the principal coordinate analysis (PCoAs) using the Bray-Curtis method, the gut microbiota of the antibiotic group diverged from that of the control group, reflecting the sensitivity of *H. halys*’ gut microbiota to SM (Figure 4A).

Moreover, a more detailed analysis of shifts in the microbiome community structure revealed significant changes at both the family and genus taxonomic levels (*p* < 0.05). At the family level, following SM treatment, the absolute abundance of Erwiniaceae (*p* = 0.03), Yersiniaceae (*p* = 0.006), and Sphingomonadaceae (*p* = 0.01) increased significantly in female *H. halys*, while that of Enterobacteriaceae (*p* = 0.009) and Xanthobacteraceae (*p* = 0.09) decreased markedly; in males, Sphingomonadaceae (*p* < 0.001) showed a significant increase, and Enterobacteriaceae (*p* = 0.01) a marked decrease. Moreover, at the genus level, the absolute abundance of *Pantoea* (*p* = 0.03), *Serratia* (*p* = 0.006), *Novosphingobium* (*p* = 0.018), and *Sphingomonas* (*p* = 0.001) increased significantly in females from the antibiotic group, whereas *Yokenella* abundance decreased markedly; in males, *Pseudacidovorax*, *Novosphingobium* and *Sphingomonas* exhibited significantly increased abundance, with *Yokenella* again showing a marked decrease (Figure 4B). Additionally, linear discriminant analysis effect size (LEFSe) was performed, which revealed that females in the antibiotic group were enriched with Proteobacteria (phylum), Gammaproteobacteria (class), Erwiniaceae (family), and *Pantoea* (genus). In contrast, males were enriched with Firmicutes (phylum), Bacilli (class), Spiroplasmataceae (family), and *Spiroplasma* (genus) (Figure 4C). These findings demonstrated that the gut bacterial community differed following antibiotic treatment relative to the control group. The balanced state of the gut microbiota contributes to maintaining body weight homeostasis, and disruption of this stability may affect the host’s utilization of nutrients and energy, thereby influencing its physiology.

### Transcriptional response of *H. halys* to SM

**Figure 5:**
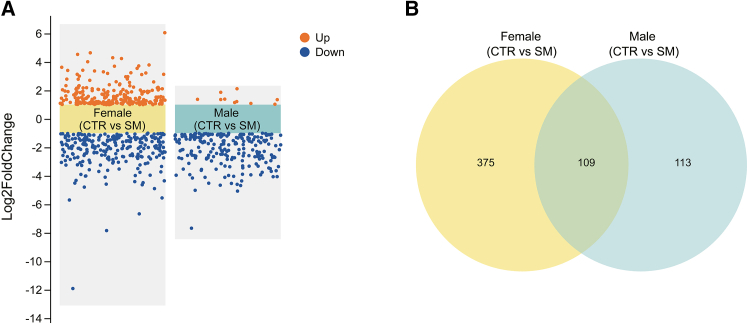
Differentially expressed transcripts in *H. halys* between the control and SM groups (A) Multi-group differential volcano plot of DEGs between the control and SM groups. The orange dots show the upregulated DEGs, and the blue dots show the downregulated DEGs (|log2-FoldChange| > 1, *p* value <0.05, *n* = 4 biological replicates per group with 7 individuals per replicate). (B) Venn diagram indicating the number of transcripts between the female and male *H. halys*.

Transcriptome analysis yielded 38–54 million raw reads per sample; after quality filtering, approximately 38–53 million clean reads (98.7%–98.9%) were retained in *H. halys* specimens (Table S7). We performed differential analysis of gene expression using DESeq, with the criteria for screening differentially expressed genes (DEGs) as follows: |log2-FoldChange|>1, *p* value <0.05. A total of 484 DEGs (237 upregulated and 247 down-regulated) and 222 DEGs (12 upregulated and 210 downregulated) were identified in female and male insects, respectively (Figure 5A). The Venn diagram shows that after SM treatment, 375 and 113 DEMs were uniquely expressed in female and male *H. halys*, respectively. In comparison, 109 DEMs were commonly expressed in both females and males (Figure 5B). We randomly selected 10 DEGs from the enriched metabolic pathways for qPCR to validate the RNA-seq data. A positive correlation was observed between the qPCR and RNA-seq results, confirming the reliability of the RNA-seq data (Figure S2).

**Figure 6:**
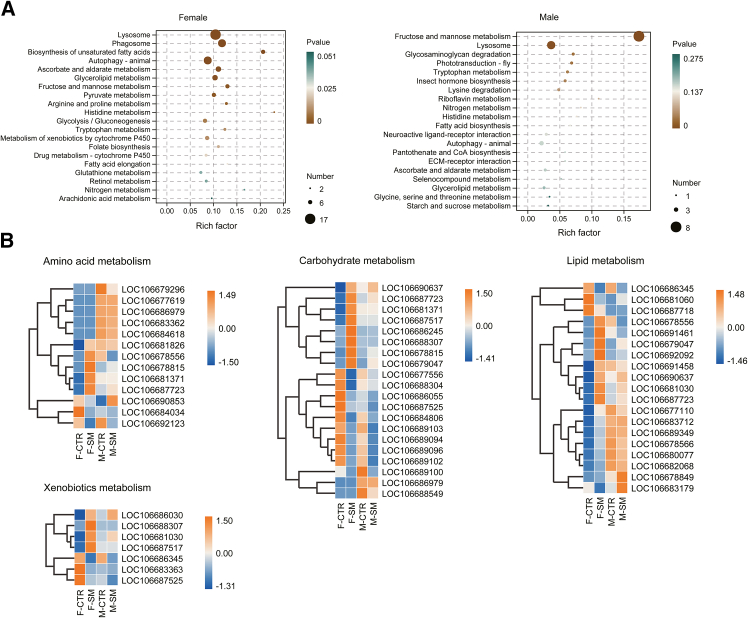
SM-induced changes in metabolic pathways and the expression profile of DEGs in key metabolic pathways (A) Top 20 differentially enriched pathways of DEGs after SM treatment based on the KEGG database. The size of the dots represents the number of DEGs annotated in the corresponding pathway, while the color’s depth indicates the statistical significance level. (B) Changes in DEGs involved in amino acid, carbohydrate, lipid, and nucleoside metabolisms under SM treatment.

To understand SM’s biological effects comprehensively, we investigated the biological significance of gene expression changes via KEGG pathway enrichment analysis. The results indicated that lipid metabolism, amino acid metabolism, carbohydrate metabolism, and metabolism of cofactors and vitamins were the most impacted pathways in female and male *H. halys* following SM treatment (Tables S8 and S9). Specifically, the DEGs in females were significantly enriched in pathways related to arginine and proline metabolism, histidine metabolism, tryptophan metabolism, ascorbate and aldarate metabolism, fructose and mannose metabolism, pyruvate metabolism, glycolysis, biosynthesis of unsaturated fatty acids, glycerolipid metabolism, and fatty acid elongation. In males, the DEGs were significantly enriched in tryptophan metabolism, fructose and mannose metabolism, and glycosaminoglycan degradation (*p* < 0.05) (Figure 6A). Several genes involved in regulating carbohydrate metabolism, amino acid metabolism, lipid metabolism, and nucleotide metabolism were significantly upregulated in females from the antibiotic group (Figure 6B); details regarding the regulation of these DEGs are provided in Tables S10 and S11.

Notably, in female *H. halys* treated with SM, the expression of aldehyde dehydrogenase (*ALDH*) and alcohol dehydrogenase (*ADH*) increased significantly. The former is involved in the metabolism of lipids, amino acids, and carbohydrates, while the latter participates in lipid and carbohydrate metabolism. In *Drosophila*, *ADH* catalyzes the oxidation of ethanol to acetaldehyde, which is then converted to acetate by *ALDH*. Acetate can be activated to acetyl-CoA and subsequently incorporated into fatty acids and lipids through *de novo* lipogenesis.^28^ The upregulation of both *ADH* and *ALDH* in SM-treated females may therefore promote the conversion of dietary carbohydrates and other substrates into lipids, contributing to increased fat storage and weight gain. In addition, the expression of elongation of very long-chain fatty acids protein (*ELO*) was upregulated in females. As a member of the very-long-chain fatty acid elongase family, this protein regulates multiple critical cellular physiological processes, including membrane composition and fluidity, lipid droplet formation, and lipid signaling pathways, by participating in the synthesis and modification of lipids.^29^ This family is also involved in the esterification of fatty acids and glycerol, which further contributes to the biosynthesis of triglycerides. In contrast, the expression pattern in male bugs treated with SM exhibited a distinct profile: The expression of glycerol-3-phosphatephosphatase (*G3PP*) was significantly upregulated, and this gene is involved in the glyoxylate and dicarboxylate metabolism pathways of *H. halys*. Previous studies have demonstrated that *G3PP* is expressed in rat adipocytes, where it facilitates glycerol production, and its expression level is substantially higher than that of glycerol kinase.^30^ Further investigations have revealed that in *Caenorhabditis elegans*, the upregulated expression of *G3PP* not only confers resistance to various stress stimuli but also extends the lifespan of nematodes and promotes their healthy aging.^31^

### Functional prediction of altered microbiomes and their associations with metabolites

**Figure 7:**
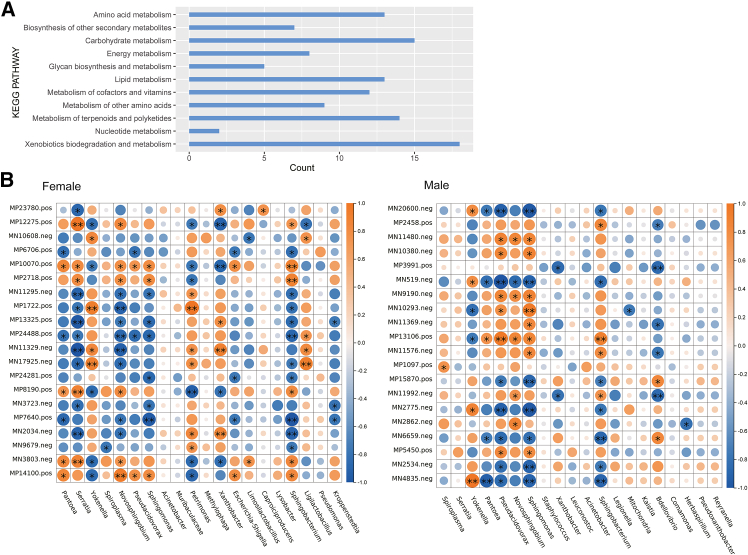
Microbes-metabolites correlations in *H. halys* (A) Statistical chart of the number of predicted KEGG secondary functional pathways, showing the functional distribution of gut microbiota across different metabolic pathways as predicted by PICRUSt2. (B) Correlation bubble heatmap of microbiota and metabolites in *H. halys.*∗ indicates significant correlations (|r| > 0.5, *p* < 0.05) between bacteria and metabolites, as determined by Spearman correlation analysis. Red circles indicate positive correlations, while blue circles denote negative correlations.

We employed phylogenetic investigation of communities by reconstruction of unobserved states (PICRUSt2) to predict the potential functions of the changes gut microbiome in *H. halys* induced by SM. Consistent with the metabolic findings, a large number of bacterial taxa were predicted to be involved in carbohydrate metabolism, amino acid metabolism, lipid metabolism, and the metabolism of cofactors and vitamins (Figure 7A). Given the extensive similarities observed in microbiome shifts, metabolite changes, and their predicted functional profiles, we conducted Spearman correlation analysis to further clarify the associations between the gut microbiome of *H. halys* and its metabolites in the antibiotic group. We observed significant correlations (|r| > 0.5, *p* < 0.05), both positive and negative, between the gut microbiome (at the genus level) and metabolites. In females, genera that increased after SM treatment, including *Pantoea*, *Serratia*, and *Sphingomonas*, were predominantly positively correlated with metabolites such as Boc-Glu-OH, protocatechuic acid, eicosapentaenoic acid, and picolinic acid, while showing negative correlations with thymidine 5′-monophosphate, cytidine 5′-monophosphate, and acetylenedicarboxylic acid. Conversely, *Yokenella*, which decreased in abundance, displayed the opposite trend: it was positively correlated with cadaverine, PG (16:1(9Z)/0:0), and cytidine 5′-monophosphate, but negatively correlated with Boc-Glu-OH, protocatechuic acid, and eicosapentaenoic acid. In males, the correlation network shifted: Genera that increased after SM treatment, including *Pseudacidovorax*, *Novosphingobium*, and *Sphingomonas*, were positively correlated with palmitic acid, 3-hydroxystearic acid, 2-oxo-eicosanoic acid, and L-malic acid, while negatively correlated with ascorbic acid, oxaloacetic acid, and chlorocarbonic acid. *Yokenella* and *Pantoea* exhibited inverse correlations with these metabolites (Figure 7B). These correlations indicate that changes in the gut microbial community induced by SM treatment affect metabolite levels in *H. halys* by influencing the metabolic functions of microorganisms, ultimately impacting the physiological status and body weight changes of *H. halys*.

### Transcriptome and metabolome correlation analysis of response to SM in *H. halys*

**Figure 8:**
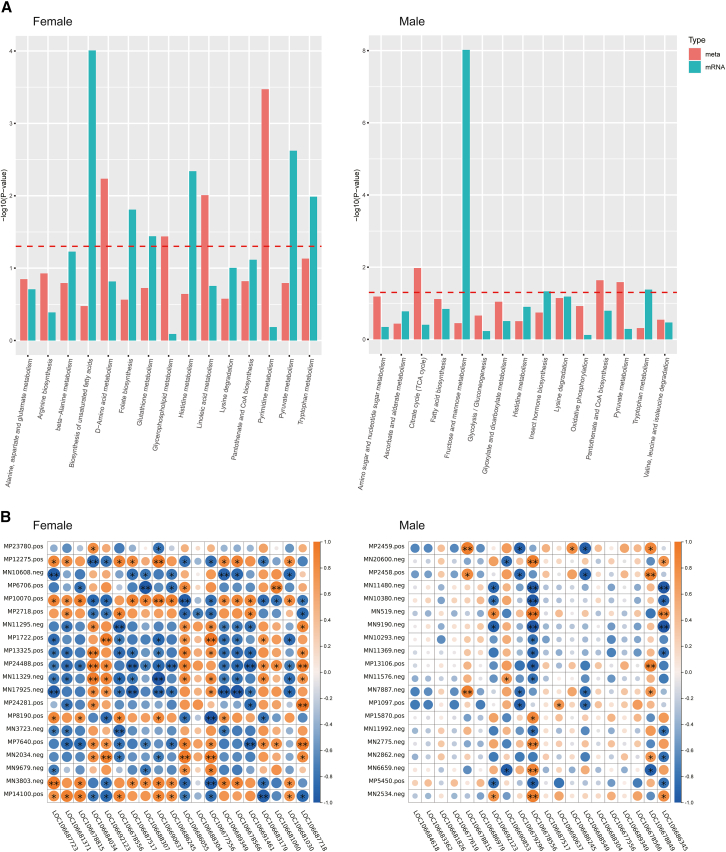
Transcriptome and metabolome correlation analysis in *H. halys* (A) KEGG pathways co-enriched by DEMs and DEGs. (B) Correlation bubble heatmap of genes and metabolites in *H. halys.* ∗ indicates significant correlations (|r| > 0.5, *p* < 0.05) between genes and metabolites, as determined by Spearman correlation analysis. Red circles indicate positive correlations, while blue circles denote negative correlations.

To clarify the response mechanism of *H. halys* to SM, this study conducted KEGG annotation and enrichment analysis of DEGs and DEMs. As shown in Figure 8A, the DEGs and differential DEMs of females were enriched in pathways such as biosynthesis of unsaturated fatty acids, D-Amino acid metabolism, pyruvate metabolism, and linoleic acid metabolism, while those of males were jointly enriched in the TCA cycle, fructose, and mannose metabolism, and pantothenate and CoA biosynthesis. Given the similarities observed in the KEGG pathways of the transcriptome and metabolome, we performed Spearman correlation analysis to further clarify the associations between differential genes and their corresponding metabolites in *H. halys* from the antibiotic-treated group. We observed significant correlations (|r| > 0.5, *p* < 0.05) between differential DEGs and DEMs. In females, genes involved in lipid and energy metabolism, such as *ALDH1A1*, *ALDH7A1*, *ADH*, and malate dehydrogenase (*MDH1*), were strongly positively correlated with eicosapentaenoic acid, protocatechuic acid, and Boc-Glu-OH, but negatively correlated with cadaverine. Conversely, D-aspartate oxidase (*DDO*) and alpha-amylase 4N (*AMY4N*) showed positive correlations with cadaverine and negative correlations with the lipid-related metabolites. In males, distinct correlation modules emerged: *MDH1* and pancreatic triacylglycerol lipase (*PTL*) were positively correlated with L-valine, palmitic acid, and L-malic acid. *G3PP* and glutathione S-transferase (*GST*) exhibited negative correlations with L-valine and L-malic acid. Mitochondrial *ALDH2* was positively correlated with chlorocarbonic acid and oxaloacetic acid, but negatively correlated with palmitic acid. These integrated analyses suggest that the transcriptional reprogramming induced by SM is functionally coupled to the observed metabolomic alterations, and the specific gene-metabolite associations point to potential regulatory nodes in the obesity-related metabolic network.

### The correlation between the body weight of *H. halys* and the three omics

**Figure 9:**
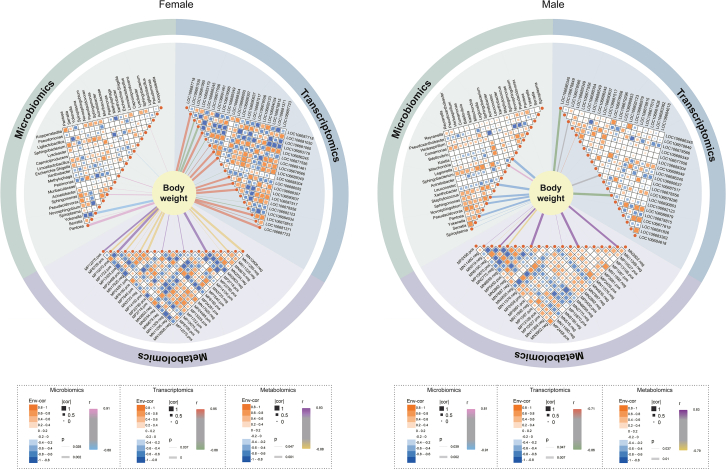
Correlation heatmap of transcriptome, metabolome, microbiome, and body weight in *H. halys* under SM treatment Green, blue, and purple represent the microbiome, transcriptome, and metabolome. The left picture shows the female *H. halys*, and the right picture shows the male *H. halys*. The pink, orange, and purple lines represent positive correlation, while the blue, green, and yellow lines represent negative correlation. Correlations were assessed using Spearman correlation analysis (|r| > 0.5, *p* < 0.05).

To investigate the correlations between body weight and multi-omics datasets (transcriptomics, metabolomics, and microbiome) in *H. halys*, we performed Spearman’s rank correlation analysis and constructed a correlation network heatmap (|r| > 0.5, *p* < 0.05). In female *H. halys*, body weight showed strong positive correlations with bacterial genera *Pantoea* and *Serratia*, while exhibiting a strong negative correlation with *Yokenella*. At the metabolic level, body weight was positively correlated with eicosapentaenoic acid and protocatechuic acid, and negatively correlated with cadaverine and cytidine-5′-monophosphate. At the transcriptional level, body weight displayed positive correlations with *PTL*, *ADH*, *ALDH1A1*, *ALDH7A1*, and a negative correlation with the carbohydrate metabolism-related gene *AMY4N*. In male *H. halys*, body weight was positively correlated with *Leuconostoc*, uridine-5′-monophosphate, and L-lysine, while negatively correlated with *ALDH2*, *GST*, and chlorocarbonic acid. These multi-omics correlations suggest that the associated microbes, metabolites, and genes are potential regulatory factors contributing to SM-induced body weight variation in *H. halys* (Figure 9).

## Discussion

The escalating obesity epidemic exerts profound impacts on both health and the economy, prompting continuous investigation into its pathogenic mechanisms.^32^^,^^33^ Although the causes of obesity are multifactorial, an imbalance between energy intake and expenditure is considered one of its core drivers.^34^ In recent years, a substantial body of research has indicated that antibiotic exposure, by reshaping the gut microbiota, can subsequently influence host energy metabolism, emerging as a significant environmental risk factor for obesity.^35^^,^^36^ Insects, as emerging model organisms for studying metabolic diseases, have had their gut microbiota widely confirmed as crucial symbiotics that regulate key physiological traits of the host.^37^ These microorganisms not only regulate host nutrient digestion, energy acquisition, metabolite synthesis, and physiological homeostasis but also engage in complex interactions with the host through various mechanisms.^38^ In the mutualistic symbiotic relationships between insects and their symbiotic bacteria, insect hosts can provide symbionts with nutrients and habitats, and may even offer direct transmission routes. In return, insects benefit from bacteria-mediated physiological processes, including protein hydrolysis, pathogen antagonism, toxic substance degradation, biological nitrogen fixation, and promotion of nutrient uptake and digestion.^39^^,^^40^ Given the central role of gut microbiota in host metabolic regulation, antibiotics, due to their profound ability to alter microbial community structure and function, have become powerful tools for studying microbe-host interaction mechanisms.^41^

**Figure 10:**
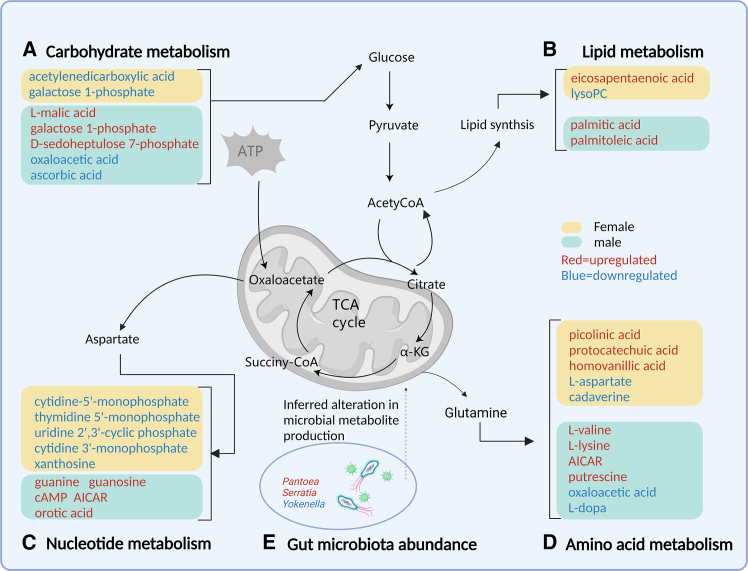
Schematic of SM-induced metabolic disorders in overall energy balance Obesity of *H. halys* (weight gain) was attributed to enhanced energy reserves (ATPs) triggered by the tricarboxylic acid (TCA) cycle that stimulated (A) carbohydrate metabolism, (B) lipid metabolism, (C) nucleotide metabolism, and (D) amino acid metabolism. (E) The SM induced changes of gut microbiota, leading to significant differences in the predicted functional profiles.

In this study, multi-omics analysis confirmed that SM treatment significantly altered the gut microbiota structure of *H. halys* while simultaneously disturbing the metabolism of carbohydrates, lipids, amino acids, and nucleotides (Figure 10). This suggests that gut microbiota remodeling may be a key mediating factor in SM-induced host metabolic disruption and weight gain.

In this study, SM treatment significantly altered the gut microbiota composition of *H. halys* at both the family and genus levels. At the family level, the relative abundances of Erwiniaceae and Enterobacteriaceae underwent marked changes. At the genus level, following SM treatment, the relative abundances of *Pantoea* and *Serratia* increased significantly in female insects, while that of *Yokenella* decreased significantly; in male insects, the abundance of *Serratia* also increased, whereas *Yokenella* abundance decreased. These differential bacterial taxa exhibit clear functional associations.

*Serratia* is a common commensal genus in the lepidopteran gut and has been reported in multiple insect species.^42^^,^^43^ In *Cinara cedri*, *Serratia symbiotica* has taken over some functions of the obligate endosymbiont *Buchnera aphidicola*, such as the synthesis of key cofactors and vitamins. Additionally, *Serratia symbiotica* is capable of utilizing acetyl-CoA to synthesize certain fatty acids, particularly the SCFA acetate.^44^^,^^45^ In *Drosophila melanogaster*, acetate produced by gut bacteria can be absorbed by the host and converted into acetyl-CoA, which then promotes lipid storage via *de novo* lipogenesis.^28^ In the present study, the increase in *Serratia* abundance following SM treatment was positively correlated with the upregulation of multiple lipid metabolites in female *H. halys*, suggesting that *Serratia* may provide SCFA precursors as substrates for host lipid synthesis, thereby promoting fat accumulation.

The enrichment of *Pantoea* also carries significant functional implications. In lepidopteran and some hemipteran insects, *Pantoea* has been reported as one of the common dominant genera of gut symbionts.^42^^,^^46^ Studies have shown that the bacterial symbiont *Pantoea carbekii* of *H. halys* encodes standard biosynthetic pathways for nearly all essential and non-essential amino acids.^27^ In hemipteran insects, gut microorganisms provide essential amino acids to the host, promoting insect development.^47^ The blueberry maggot fly can establish a mutualistic symbiotic relationship with *Pantoea agglomerans*, a bacterium that mediates the key nitrogen cycling process in the host intestinal tract, while the blueberry maggot fly provides it with nutrients and a suitable living habitat.^40^ Therefore, the enrichment of *Pantoea* following SM treatment likely enhanced the amino acid supply capacity of the gut microbiota, providing the host with a more abundant substrate for protein synthesis, thereby promoting host protein synthesis and weight gain. This interpretation is highly consistent with our metabolomics results: the levels of multiple amino acids (e.g., L-valine, L-lysine) were significantly upregulated in the SM-treated group, and these amino acids were positively correlated with *Pantoea* abundance.

Different bacterial taxa each perform specific biological functions, so alterations in the structure of the gut microbiota are often accompanied by changes in its overall functional capacity.^48^ Functional prediction analysis revealed that SM treatment significantly modified the functional potential of the gut microbiota in *H. halys* concerning carbohydrate metabolism, amino acid metabolism, lipid metabolism, and cofactor and vitamin metabolism, which was highly consistent with the observed changes in the metabolite profiles. Gut microbiota dysbiosis is typically associated with metabolic disturbances, thereby affecting the overall health status and physiological functions of the host. Existing studies have shown that bactericidal antibiotics can directly alter bacterial cellular metabolic processes. Vancomycin treatment significantly altered the structure and metabolic function of the gut microbiota in mice, as evidenced by reductions in metabolites such as SCFAs and amino acids, as well as a decrease in host-microbiota co-metabolites (e.g., hippurate).^49^^,^^50^ Furthermore, vitamin B12 produced by the intestinal microbiota influences the development and fecundity of *Caenorhabditis elegans*.^51^ A seven-day antibiotic exposure affected microbial carbohydrate metabolism in the intestine of *Reticulitermes grassei*.^52^

Carbohydrates, lipids, and amino acids represent the three primary energy reserves required for organismal growth, development, and other physiological processes.^53^^,^^54^ Among these, lipids play a crucial role in insect life, providing energy for growth, development, and other essential processes such as flight, migration, diapause, and pheromone synthesis.^55^ Our KEGG analysis indicated that SM treatment affected the expression of genes related to lipid metabolism. Specifically, the pathways involved in female *H. halys* included linoleic acid metabolism, glycerophospholipid metabolism, and biosynthesis of unsaturated fatty acids; this maintained energy reserves for physiological processes and provided energy for the growth of *H. halys*. In the SM-treated group of female *H. halys*, the level of the metabolite eicosapentaenoic acid was upregulated, while the level of lysoPC (16:1(9Z)) was downregulated. Eicosapentaenoic acid is an essential polyunsaturated fatty acid, and its upregulation may promote fat accumulation. In predatory insects such as *Bythotrephes longimanus*, consuming food rich in eicosapentaenoic acid significantly increases body weight and fecundity.^56^ When eicosapentaenoic acid was added to the diet of *Tenebrio molitor* larvae, the glycerophospholipid metabolism pathway was activated, which converted eicosapentaenoic acid into medium-chain and short-chain phospholipids with higher bioavailability.^57^ LysoPC is an important cellular signaling molecule that can transport glycerophospholipid components such as fatty acids, phosphatidylglycerol, and choline between tissues.^58^ In terrestrial animals, lysophospholipids are commonly used to enhance lipid digestion and absorption;^59^^,^^60^ in mouse studies, the disruption of the balance between phosphatidylcholine and lysoPC leads to abnormal lipid metabolism.^61^ The lipid metabolism-related pathways involved in male *H. halys* included fatty acid biosynthesis, fatty acid elongation, and biosynthesis of unsaturated fatty acids, among others. The levels of palmitic acid and palmitoleic acid were significantly upregulated in the SM-treated group, and these two fatty acids are jointly involved in fatty acid biosynthesis. Palmitic acid is a type of saturated fatty acid, which can be obtained not only through dietary intake but also synthesized endogenously from other fatty acids, carbohydrates, and amino acids.^62^ Studies have demonstrated that palmitic acid can induce inflammatory responses and glucose metabolism disorders.^63^ In mice fed a high-fat diet, the serum palmitic acid content and the expression levels of inflammation-related factors were both significantly increased, while glucose tolerance and insulin sensitivity were markedly impaired.^64^ Palm oil has relatively high contents of palmitoleic acid (accounting for 44%) and palmitic acid (accounting for 37%). Excessive intake of palm oil leads to more significant weight gain, decreased survival rate, elevated blood glucose levels, and concomitant fat accumulation in honeybees.^65^ These changes in lipid metabolites may result in increased fat synthesis and enhanced energy storage in *H. halys*, thereby contributing to weight gain.

In insects, carbohydrate metabolism occurs throughout the entire life cycle and plays essential roles in energy supply and growth support.^66^ SM treatment upregulated genes involved in the TCA cycle, fructose and mannose metabolism, and glycolysis, promoting carbohydrate and energy metabolism in *H. halys*. In the SM-treated group of male *H. halys*, the metabolites L-malic acid and galactose 1-phosphate were upregulated, while oxaloacetic acid and ascorbic acid were downregulated. These changes in carbohydrate metabolites may affect energy supply and utilization efficiency in *H. halys*. L-malic acid is an important organic acid produced during organismal metabolism and serves as a key intermediate in the TCA cycle and its branch, the glyoxylate cycle.^67^ It can promote intracellular ATP production, enhance energy supply, and facilitate growth, development, and weight gain.^68^ Oxaloacetic acid plays a vital role in regulating the TCA cycle, gluconeogenesis, urea cycle, and amino acid synthesis;^69^ its downregulation may disrupt the normal progression of energy metabolism.

Additionally, SM treatment upregulated genes associated with amino acid metabolism. KEGG results showed that SM treatment upregulated genes involved in the metabolism of arginine, proline, and tryptophan, as well as genes related to the biosynthesis of valine and leucine. The fundamental function of amino acids is to synthesize proteins in the body;^70^ they also act as bioactive molecules and exert non-proteinogenic functions in nutrient metabolism, stress responses, and tissue development.^71^ These findings suggest that SM-induced microbial changes disrupt metabolic balance in the body and enhance energy reserves, which may represent the primary mechanism underlying weight gain in *H. halys*.

In summary, this study demonstrates that SM-induced gut microbiota dysbiosis can ultimately lead to weight gain in *H. halys* by disrupting host metabolic balance and enhancing energy reserves. Following SM treatment, the enhanced functional capacity of the gut microbiota in carbohydrate, amino acid, lipid, and cofactor metabolism supported the efficient operation of host energy metabolism and anabolic processes, thereby creating a stable physiological environment for weight gain. These findings are highly consistent with previous research conclusions that antibiotics influence host metabolism by reshaping the gut microbiota, further confirming the central role of gut microbes in mediating the metabolic effects of antibiotics. However, the specific mechanisms through which the gut microbiota of *H. halys* exerts its effects within the host remain to be elucidated. Future studies should employ germ-free model construction, microbiota transplantation, and functional gene validation to verify the functional roles of key obesity-related bacteria and establish causal relationships between them and host metabolic changes. Secondly, given the observed sex-specific responses, future research should investigate the hormonal or genetic mechanisms underlying these differences. Finally, the possibility of targeting the gut microbiome to modulate the metabolic health of agricultural pests or beneficial insects could be explored.

### Limitations of the study

While this study provides multi-omics insights into SM-induced obesity in *H. halys*, several limitations should be acknowledged. First, our study predominantly relied on a single laboratory-reared colony of *H. halys*. While this controls for genetic background, it does not account for potential variability in response to SM that might exist across different natural populations. Second, the functional predictions of the gut microbiome via PICRUSt2, while informative and consistent with our metabolomic and transcriptomic data, are inferred rather than directly measured. Finally, while our integrated analysis reveals strong correlations between the gut microbiome, metabolome, and transcriptome, it establishes associations rather than definitive causal relationships. The specific molecular mechanisms by which key bacterial taxa (e.g., *Pantoea*, *Serratia*) directly drive the observed metabolic and transcriptional changes in the host remain to be experimentally validated through targeted interventions such as microbiota transplantation or gene knockout studies.

## Resource availability

### Lead contact

Further information and requests for resources and reagents should be directed to and will be fulfilled by the lead contact, Chenxi Liu (liuchenxi@caas.cn).

### Materials availability

This study did not generate new unique reagents.

### Data and code availability

• Sequencing data generated in this study have been deposited in the NCBI BioProject database under accession nos. PRJNA1321010 (RNA-seq) and PRJNA1370607 (16S rRNA). These data are also listed in the key resources table.
• This paper does not report original code. All other datasets are available from the lead contact upon request.

## Acknowledgments

This study was supported by the 10.13039/501100000943Commonwealth Scientific and Industrial Research Organisation, CSIRO [2021010026, 2024082216]. The sponsors played no role in any aspect of this study.

## Author contributions

Conceptualization, X.Y. and C.L.; methodology, X.Y., D.L., and Y.C.; investigation, X.Y. and Y.L.; validation, X.Y., X.D., and C.L.; writing – original draft, X.Y.; writing – review and editing, W.X., Z.S., X.D., and C.L.; funding acquisition and resources, C.L.; supervision, X.D. and C.L.

## Declaration of interests

The authors declare no competing interests.

## STAR★Methods

### Key resources table

**Table:** 

REAGENT or RESOURCE	SOURCE	IDENTIFIER
**Biological samples**		

*H. halys* gut tissue	Laboratory-reared colony, CAAS	N/A

**Chemicals, peptides, and recombinant proteins**		

Streptomycin sulfate	Amresco, USA	CAS: 3810-74-0

**Critical commercial assays**		

OMEGA Soil DNA Kit	Omega Bio-Tek, USA	Cat# D5625-01
TruSeq RNA Sample Preparation Kit	Illumina, USA	Cat# RS-122-2001
T5 Fast qPCR Mix	TSINGKE Biotech Co., China	Cat# TSE201

**Deposited data**		

Raw RNA sequencing data	NCBI BioProject	PRJNA1321010
16S rRNA data	NCBI BioProject	PRJNA1370607
*H. halys* reference genome	NCBI	GCF_Hhal_2.0

**Experimental models: Organisms/strains**		

*H. halys* (brown marmorated stink bug)	Laboratory-reared colony, CAAS	N/A

**Oligonucleotides**		

16S rRNA primer 338F: 5'-ACTCCTACGGGAGGCAGCA-3'	This study	N/A
16S rRNA primer 806R: 5'-GGACTACHVGGGTWTCTAAT-3'	This study	N/A
GAPDH-F (qPCR): TCCCCTTTCGAGCAGGTATG	This study	N/A
GAPDH-R (qPCR): AGCCTTTTCGCAGGTTTATGAG	This study	N/A

**Software and algorithms**		

DESeq (v1.38.3)	Bioconductor	https://bioconductor.org/packages/DESeq
PICRUSt2	GitHub	https://github.com/picrust/picrust2

### Experimental model and study participant details

*H. halys* population in this study was originally initiated from adult specimens collected from field sites in Sanming City, Fujian Province, China. Experimental individuals were obtained from a laboratory-reared colony that has been sustained for more than 35 generations, with corn as the dietary source and rearing conducted in boxes (34.5×23.3×16 cm). The insects were cultured under controlled environmental conditions: a temperature of 26±1°C, a photoperiod of 16 h light/8 h dark, and a relative humidity of 65±5%. Adult individuals aged 1–3 days postemergence were selected for the experiments.

### Method details

#### Antibiotic treatment

SM (CAS: 3810-74-0, Amresco, USA) was respectively diluted to 0.1 g/L, 0.6 g/L and 1.0 g/L in deionized water. Corn and cotton balls were soaked in the configured solution for 2 h before feeding *H. halys* aged 1–3 days postemergence for 14 days; the control group was fed with corn and cotton balls soaked in deionized water.

#### Individual indices measurement

For body weight measurement, the control and treatment groups were weighed with a microbalance (Sartorius BT125D). Each group consisted of 400 individuals (200 males and 200 females). The number of dead and surviving individuals in each group was recorded throughout the experimental period to monitor survival rates.

#### Gut tissue collection

*H. halys* were subjected to surface sterilization with 70% ethanol for 5 min, after which they were rinsed three times with sterile water. Thereafter, the insects were soaked in pre-cooled phosphate-buffered saline (PBS) and dissected on ice using sterile forceps within a sterile workspace. Isolated gut tissues were rinsed in sterile PBS for 3 min and then transferred to 1.5 mL Eppendorf tubes. All collected samples were promptly frozen in liquid nitrogen and stored at −80°C until subsequent use. Each sample contained guts from 40 adults, and six biological replicates were prepared for each treatment group (control and antibiotic treatment).

#### Identification of intestinal bacteria

Genomic DNA was extracted from all samples using the OMEGA Soil DNA Kit (Omega Bio-Tek, USA) following the manufacturer’s recommended protocol. The quality and integrity of the extracted DNA were evaluated by agarose gel electrophoresis. Meanwhile, DNA concentration was quantified with a NanoDrop 2000 (Thermo Scientific, USA). The V3--V4 hypervariable region of the bacterial 16S rRNA gene was amplified by polymerase chain reaction (PCR) with the forward primer 338F (5’-ACTCCTACGGGAGGCAGCA-3’) and reverse primer 806R (5’-GGACTACHVGGGTWTCTAAT-3’). Each primer was tagged with a unique 7 bp sample-specific barcode to enable multiplex sequencing. The 25 μL PCR reaction system consisted of 5 μL of 5× buffer, 2 μL of 2.5 mM dNTPs, 0.25 μL of 5 U/μL Fast pfu DNA polymerase, 1 μL of DNA template, 1 μL each of 10 μM forward and reverse primers, and ddH2O to bring the volume to the final 25 μL. The thermal cycling program was set as follows: initial denaturation at 98°C for 5 min, followed by 25 cycles of 98°C for 30 s (denaturation), 53°C for 30 s (annealing), and 72°C for 45 s (extension), with a final extension step at 72°C for 5 min. PCR amplicons were quantified with a Quant-iT PicoGreen dsDNA assay kit (Invitrogen, USA), while purification was carried out using Vazyme VAHTSTM DNA clean beads (Vazyme, China). Fluorescence quantification was performed on the PCR-amplified and recovered products using the Quant-iT PicoGreen dsDNA Assay Kit, with a microplate reader (BioTek, FLx800) serving as the quantification instrument. Based on the fluorescence quantification results, the products of each sample were mixed in corresponding proportions according to the required sequencing depth for individual samples, and high-throughput sequencing was finally conducted on the Illumina NovaSeq PE250 platform.

#### RNA-seq analysis

Seven individuals of *H. halys* were selected from each treatment group, with four replicates per group. Total RNA was isolated from each group using the TRIzol reagent kit (Invitrogen, USA), with genomic DNA eliminated via digestion with DNase I (Fermentas, Canada). RNA concentration was determined using a NanoDrop 2000 spectrophotometer (Thermo Scientific, USA), while RNA purity and integrity were evaluated with an Agilent 2100 Bioanalyzer (Agilent, USA). Samples meeting the criteria of A260/280 ratio (absorbance at 260 nm versus 280 nm) ≥ 1.8 and RNA integrity number (RIN) ≥ 7 were selected for subsequent library construction and sequencing. Following the manufacturer’s instructions, 1 μg of total RNA per sample was utilized for cDNA library preparation with the TruSeq RNA Sample Preparation Kit (Illumina, USA). mRNA was first enriched using oligo (dT) magnetic beads, followed by fragmentation through the addition of fragmentation buffer. Double-stranded cDNA was synthesized using random hexamer primers. Subsequently, the resulting cDNA underwent end repair, A-base addition, and ligation. Target cDNA fragments of 400 bp were isolated by AMPure XP beads (Beckman Coulter, USA), followed by PCR amplification for 15 PCR cycles. After quantification, Shanghai Personal Biotechnology performed the sequencing on the NovaSeq 6000 platform. We aligned reads from all the samples to the reference *H. halys* genome (NCBI: GCF_Hhal_2.0) using the HISAT2 package.

Raw sequencing data were stored in FASTQ format, and the original sequencing datasets have been submitted to the NCBI SRA (PRJNA1321010). HTSeq software (v0.9.1) was utilized to map Read Count values to individual genes, corresponding to the initial gene expression levels. Subsequently, we used FPKM to standardize the expression. Differential gene expression analysis was performed using DESeq (v1.38.3) with the screening criteria: absolute value of log2 fold change (|log2FoldChange|) > 1 and statistical significance threshold of P-value < 0.05. Finally, we conducted Kyoto Encyclopedia of Genes and Genomes (KEGG) pathways using clusterProfiler (v4.6.0) software. In all bioinformatic analyses, a P-value < 0.05 was defined as statistically significant enrichment.

#### Quantitative real-time PCR (qPCR)

qRT-PCR was performed on a QuantStudio 5 thermocycler (Applied Biosystems, USA), with a total reaction volume of 20 μL. The reaction mixture contained 2 μL of cDNA template, 10 μL of 2× T5 Fast qPCR Mix (TSINGKE Biotech Co., China), 0.8 μL of forward primer, 0.8 μL of reverse primer, 0.4 μL of 50× ROX Reference Dye I, and 6 μL of nuclease-free water. The thermal cycling program was as follows: initial denaturation at 95°C for 1 min, followed by 40 cycles of 95°C for 10 s (denaturation), 60°C for 5 s (annealing), and 72°C for 10 s (extension). The relative mRNA expression levels were normalized to the housekeeping gene GAPDH of *H. halys* (forward primer: TCCCCTTTCGAGCAGGTATG; reverse primer: AGCCTTTTCGCAGGTTTATGAG) and quantified using the 2−ΔΔCt method.^72^ All experiments were performed with at least three independent biological replicates, and the representative results are shown. Detailed information on all primers utilizedin this study is provided in Table S1.

#### Untargeted metabolomics analysis

Seven individuals of *H. halys* were selected from each treatment group, with six replicates per group. Each sample was mixed with 200 μL of pre-cooled ultrapure water and two steel beads, then placed in a high-throughput tissue grinder for homogenization at 55 Hz for 60 s. Subsequently, 800 μL of methanol:acetonitrile (1:1, v/v) was added to the homogenate. The mixture was sonicated in an ultrasonic cleaner for 30 minutes, then frozen at -20°C for 30 minutes. After centrifugation at 12,000 rpm for 10 min at 4°C, 800 μL of the supernatant was collected and filtered through a 0.22 μm filter. The filtered samples were analyzed using a liquid chromatography-tandem mass spectrometer (LC-MS). An ACQUITY UPLC HSS T3 column (100Å, 1.8 μm, 2.1 mm × 100 mm) was utilized, with the following parameters: flow rate of 0.4 mL/min, column temperature maintained at 40°C, autosampler temperature set at 8°C, and injection volume of 2 μL. Gradient elution was implemented using mobile phase A (0.1% formic acid aqueous solution) and mobile phase B (acetonitrile supplemented with 0.1% formic acid). A heated electrospray ionization (HESI) source was adopted, and mass spectrometry data were acquired in both positive and negative ion modes using a Thermo Orbitrap Exploris 120 mass spectrometer, which was controlled by Xcalibur software (version 4.7, Thermo Scientific, USA).

The commercial software Compound Discoverer™ 3.3 (version 3.3.2.31, Thermo, USA) was used for peak extraction, peak alignment, and quantitative analysis of the acquired mass spectrometry data. Only ion peaks with a relative standard deviation (RSD) below 30% in quality control (QC) samples were retained for subsequent analysis. Metabolite identification was accomplished by matching against multiple databases, including a self-constructed library (PSNGM Database), the online mzCloud library (https://www.mzcloud.org/), LIPID MAPS (https://www.lipidmaps.org/), the Human Metabolome Database (HMDB, https://hmdb.ca/), MassBank of North America (MoNA, https://mona.fiehnlab.ucdavis.edu/), and the NIST_2020_MSMS spectral library. The R package Ropls was employed to conduct dimensionality reduction analyses on the sample data, including principal component analysis (PCA), partial least squares discriminant analysis (PLS-DA), and orthogonal partial least squares discriminant analysis (OPLS-DA), each performed independently. Differentially expressed metabolites (DEMs) were screened using the criteria of P-value < 0.05 and variable importance in projection (VIP) > 1, followed by KEGG pathway enrichment analysis.

### Quantification and statistical analysis

To compare the differences between the control and antibiotic groups in terms of body weight, metabolite profiles, absolute abundance of gut microbiota, and changes in gene expression, the independent samples t-test was performed. Alpha and beta diversity indices were computed using relevant R packages. Potential functional profiles of the SM-induced gut changes in *H. halys* were predicted using the phylogenetic Investigation of Communities by Reconstruction of Unobserved States (PICRUSt2) analysis. Spearman’s correlation analysis was conducted between the gut microbiome and metabolites, as well as between transcriptome and metabolome, with a correlation coefficient |r| > 0.5 and p < 0.05 considered significant. Statistical significance was identified with ∗p < 0.05; ∗∗p < 0.01; and ns, not significant. All error bars represent mean ± SEM.

## Supplemental information

Document S1. Figures S1 and S2

Table S1. Primers of the selected genes for quantitative real-time PCR

Table S2. SM-induced differential metabolites in female *H. halys*

Table S3. SM-induced differential metabolites in male *H. halys*

Table S4. KEGG pathways involved in differential metabolites of female *H. halys* under SM treatment

Table S5. KEGG pathways involved in differential metabolites of male *H. halys* under SM treatment

Table S6. Summary of gut microbiota data in different treatments of *H. halys*

Table S7. Quality control and statistics of transcriptomic data

Table S8. KEGG pathway enrichment analysis of female DEGs in *H. halys* post SM Treatment

Table S9. KEGG pathway enrichment analysis of male DEGs in *H. halys* post SM Treatment

Table S10. SM-induced differentially expressed genes in female *H. halys*

Table S11. SM-induced differentially expressed genes in male *H. halys*
