# Supplementary material for: Multi-omics reveals that streptomycin sulfate induces obesity in Halyomorpha halys by disrupting the gut microbiome-metabolome axis
Source: iScience. 2026 Jun 30;29(7):116612. doi: 10.1016/j.isci.2026.116612 (PMC13378138; doi:10.1016/j.isci.2026.116612)
Supplement: Document S1. Figures S1 and S2 [file mmc1.pdf]

iScience, Volume *29*

Supplemental information

Multi-omics reveals that streptomycin sulfate induces obesity in *Halyomorpha halys* by disrupting the gut microbiome-metabolome axis

Xiaoyu Yan, Yaquan Lv, Dianyu Liu, Yu Chen, Zhihan Su, Wenyan Xu, Xiaolin Dong, and Chenxi Liu

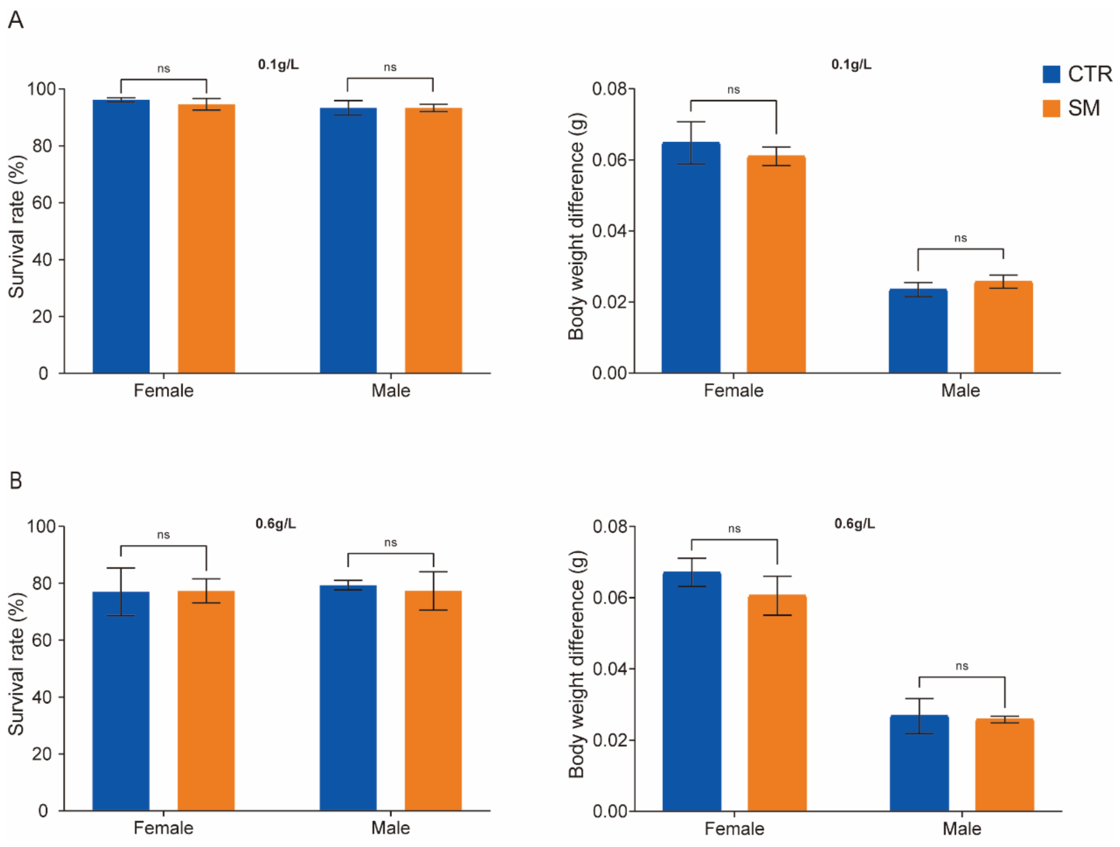

Figure S1. Effects of SM on survival rate and body weight of *H. halys* of different sexes.

(A) 0.1g/L SM on survival rate and body weight of *H. halys* (n = 200 individuals per sex per group).

(B) 0.6g/L SM on survival rate and body weight of *H. halys* (n = 200 individuals per sex per group).

Error bars indicate standard error of the mean (SEM); Statistical significance was identified with $*p < 0.05$; $**p < 0.01$; and ns, not significant. CTR: control group. SM: streptomycin sulfate-treated group.

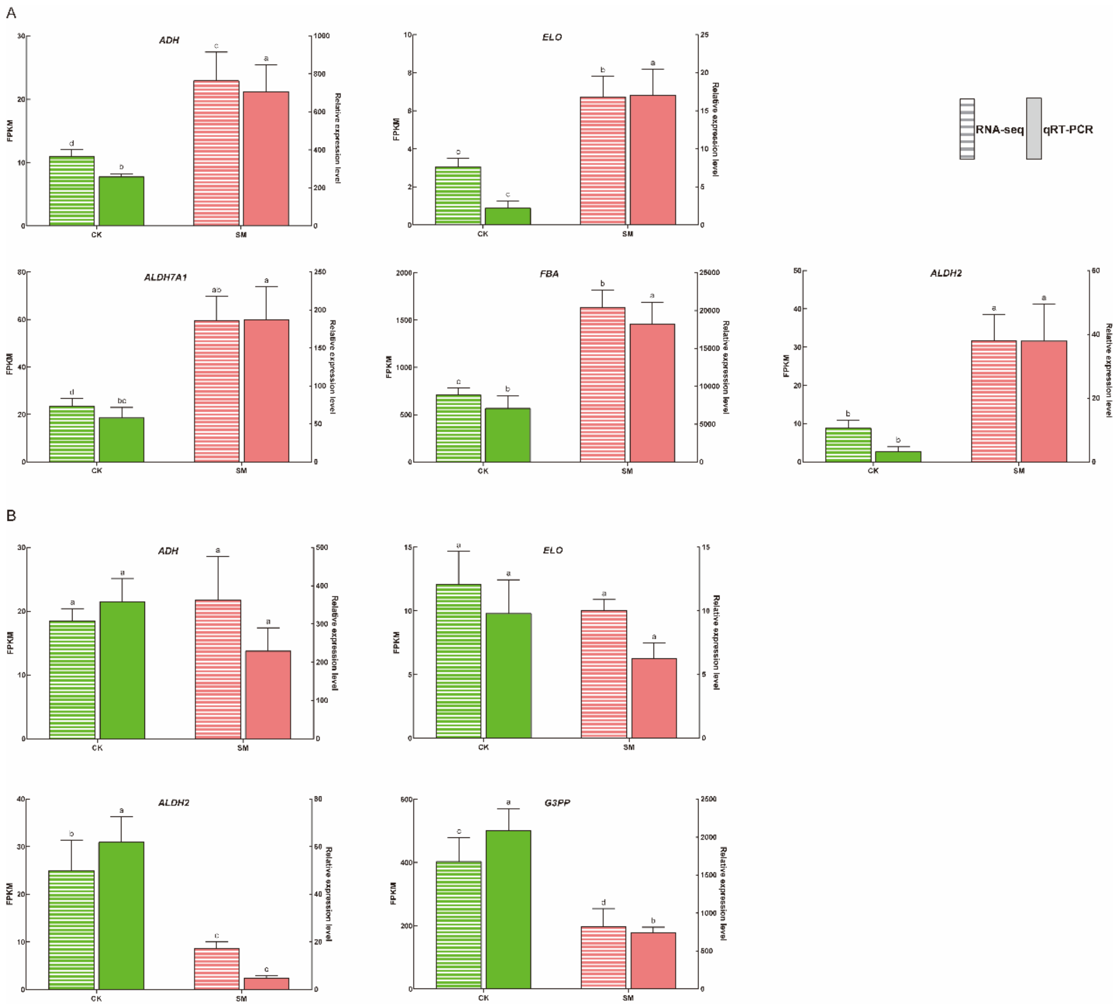

Figure S2. Validation of RNA-seq data by qPCR.

(A) Comparison of gene expression data between RNA-seq and qPCR in female *H. halys* under 1 g/L SM Treatment.

(B) Comparison of gene expression data between RNA-seq and qPCR in male *H. halys* under 1 g/L SM Treatment. Data are represented as mean ± SEM. Different lowercase letters (a, b, c, d) indicate significant differences between groups ($p < 0.05$, one-way ANOVA followed by Tukey's HSD post hoc test). Groups sharing the same letter are not significantly different.
